# Estimation of salivary matrix metalloproteinases- 12 (MMP- 12) levels among patients presenting with oral submucous fibrosis and oral squamous cell carcinoma

**DOI:** 10.1186/s12903-021-01571-7

**Published:** 2021-04-23

**Authors:** Zohra Saleem, Abdul Hafeez Shaikh, Uzma Zaman, Shaheen Ahmed, Muhammad Mansoor Majeed, Anum Kazmi, Waqas Ahmed Farooqui

**Affiliations:** 1 Department of Oral and Maxillofacial Surgery, Dow University of Health Sciences, Karachi, Pakistan; 2 Department of Biochemistry, Dow University of Health Sciences, Karachi, Pakistan; 3 Department of Oral Biology, Altamash Institute of Dental Medicine, Karachi, Pakistan; 4 School of Public Health, Dow University of Health Sciences, Karachi, Pakistan

**Keywords:** Matrix metalloproteinase, MMP-12, Oral squamous cell carcinoma, Oral submucous fibrosis, Salivary biomarker, ELISA

## Abstract

**Background:**

Oral squamous cell carcinoma is a global threat and accounts for approximately 90% of malignant oral lesions. The emergence of oral carcinoma is linked to precancerous lesions, which act as precursors of the disease. Matrix metalloproteinases appear to play a significant role in the pathogenesis of both precancerous conditions and oral malignancies due to their participation in remodeling of the extracellular matrix.

**Methodology:**

This is an analytical study conducted at Dow University of Health Sciences, Pakistan. Unstimulated saliva samples were collected from healthy, oral submucous fibrosis and oral squamous cell carcinoma patients. The level of MMP-12 was estimated using enzyme-linked immunosorbent assay. One-way Analysis of variance was run to determine if MMP-12 levels differ between the three groups, which was preceded by post hoc Tuckey test. MMP-12 cut off values were determined using Receiver operating characteristic curve.

**Results:**

A significant difference in salivary MMP-12 expression was observed in OSF and OSCC (*p* < 0.001). The expression of salivary MMP-12 was higher in OSF and OSCC patients as compared to the healthy group (*p* < 0.001). The mean MMP-12 expression in OSCC appeared higher than in OSF cases (*p* < 0.05). MMP-12 value of $\ge$ 4.05 ng/ml and $\ge$ 4.20 ng/ml is predictive of OSF and OSCC respectively, with 100% sensitivity and specificity (*p* < 0.001).

**Conclusion:**

Increased expression of MMP-12 appears as the healthy patient advances to OSF and OSCC. The study results also demonstrate higher MMP-12 expression in OSCC patients as compared to OSF. Therefore, the estimation of salivary MMP-12 serves as a valuable non-invasive early diagnostic tool in diagnosing oral submucous fibrosis and oral squamous cell carcinoma.

## Background

Oral cancer poses a significant health threat worldwide with noteworthy incidence and mortality in developing countries [1]. Despite an increase in the knowledge of disease prevention and treatment, an increasing number of cases every year is quite evident. Oral cavity cancers appear to be among the most prevalent cancers globally and occur in nearly one-fifth of all cancers in males and one-tenth of all cancers in females globally [2].

The emergence of oral carcinoma is linked to precancerous lesions, which act as precursors of the disease [3]. These precancerous lesions include erythroplakia, leukoplakia, and oral submucous fibrosis (OSF), with oral submucous fibrous having high potential for malignant transformation among people residing in south and southeast Asia [4]. The rate of transformation of oral submucous fibrosis to oral squamous cell carcinoma (OSCC) is roughly around 2.3–7.6% [5]. The risk is increased in patients consuming heavy tobacco and alcohol [6]. Early and correct diagnosis of highly suspicious precancerous lesions helps in timely treatment and prevents transformation into malignancy. Tumor responds well to treatment modalities in the early stage as compared to the late stage. This is evident by the outcome that approximately 80% of patients have an expected 5- years of survival [7].

Histological examination is the gold standard but, to some extent, not practicable because of the nature and therapeutic response of the tumor [8]. It has a major drawback of being an invasive method as well as painful and time-consuming [9, 10]. Therefore, the focus is being directed towards non-invasive methods for the detection of oral squamous cell carcinoma. Changes in human genetics can be identified in patient's body fluid like saliva, cerebrospinal fluid, blood serum, and urine. These body fluids reflect an alteration in proteins and nucleic acid and therefore can be considered effective biomarkers for detection of oral squamous cell carcinoma at an early stage [11].

Until now, several oral salivary biomarkers have been studied for different premalignant lesions like oral lichen planus [12], leukoplakia [13], OSF [14], but not enough data is available for a biomarker that could aid in primary OSCC detection at the non-detectable stage or precursor stage [15]. Identifying a biomarker may serve threefold benefits; detection of the tumor at an early stage, serving as a prognostic marker, and a therapeutic target [15]. Matrix metalloproteinase (MMPs) are considered one of those potential biomarkers that can theoretically fulfil all these purposes. It has been suggested that estimation of MMPs levels in various tissue fluids serve as an accessible, non-time-consuming, and non-invasive tool for diagnosis of primary disease along with subsequent prognostic monitoring [15].

Several studies observed MMPs expression in oral cancer and demonstrated enhanced expression of different MMPs and advancement of the disease as compared to controls. Generally, increased expression of MMP-1, MMP-2, MMP-3, MMP-7, MMP-9, MMP-13, and MMP-14 are associated with cancer advancement, which relates to poor cell differentiation, tumor invasion, distant metastasis, and poor prognosis [15]. The protein concentrations of MMP-1, MMP-2, MMP-3, and MMP-9 also have been seen to be greater in OSCC tumor tissue in contrast to control tissue [16].

The aim and objective of this study is to estimate and compare the levels of salivary MMP-12 in patients presenting with oral submucous fibrosis and patients presenting with oral squamous cell carcinoma.

## Methodology

### Study design and setting

It is a descriptive & analytical cross-sectional study. The study was conducted on 90 randomly selected participants presenting to Oral and maxillofacial surgery departments of Dow University of health sciences, Karachi, after obtaining permission from Institutional Review Board (IRB- 1322/DUHS/Approval/2019/71).

### Sample size

The sample size of 6 subjects per group was calculated using PASS version 15 software, based on two sample T test, allowing unequal variance with 99% Confidence of interval and 99% power of test, $\pm$ SD of MMP-12 in healthy subjects (0) and in OSCC (0.365 $\pm$ 0.113) [17]. Keeping in view low subjects in each group, we increased the sample size to 90 subjects (30 per group).

### Study participants

There were three groups of patients contributing to the study. Each group consisted of an equal number of participants (30 participants), selected randomly based on inclusion and exclusion criteria. Group 1 represented healthy individuals who demonstrated an absence of clinical and radiographic manifestation of precancerous condition and oral carcinoma and answering 'no' to dryness- related questions. Group 2 consisted of patients presenting with clinical evidence of oral submucous fibrosis, and group 3 represented patients presenting to the OPD with biopsy-proven cases of oral squamous cell carcinoma regardless of any grade.

Participants for the study were enrolled after performing a thorough oral examination, including assessment of oral mucosa, intra- oral bony areas, teeth, and periodontal tissues. Patients presenting with uncontrolled systemic diseases (uncontrolled diabetes/ hypertension), periodontal disease, salivary gland pathology, or autoimmune diseases such as SLE, rheumatoid arthritis, or Sjogren's syndrome were excluded from the study. We also excluded patients reporting acute or chronic use of drugs known to induce oral dryness, including antidepressants, antipsychotics, and antihistamines.

### Study questionnaire

The previously validated OSF and OSCC patients' questionnaire is department-designed (OMFS department, Dow University of Health Sciences). It has been pretested on several patients for the past few years for maintaining the record. The first part of the questionnaire contained questions related to socio-demographic data. The second part of the questionnaire contained questions about disease status.

The Performa for healthy participants was filled out to facilitate whether the participant qualifies the inclusion criteria. The healthy participants included students, OPD assistants, friends, and family members. The questionnaire consists of five parts: the patient's demographic features and personal information, medical history, tobacco and alcohol habits, oral hygiene practice, and other information related to oral dryness [18].

Informed consent in accordance with Helsinki's declaration was sought from all participants, and assurance of confidentiality about personal data was provided.

### Saliva collection & quantitative assessment by ELISA

For saliva sample collection, patients were seated in a clean and comfortable environment with the dental chair in an upright position. They were advised not to communicate during the procedure and not to forcefully spit or cough up mucus but let the saliva drool in 15 ml Falcon tubes once collected on the floor of the mouth. Around 2-5 ml of unstimulated saliva was gathered in a falcon tube by passive drooling method, which is considered an assuring alternative for minimizing potential error sources [18]. The effect of possible environmental factors (tobacco chewing, betel nut chewing, smokeless tobacco consumption, smoking) was controlled at the analysis phase through Analysis of covariance (ANCOVA).

Collected saliva was centrifugated at 6000×*g* at 4 °C for 10 min, and the supernatant obtained was carefully transferred equally into 3 Eppendorf tubes (microcentrifuge tubes- 1 ml) via pipette Juster. After disinfection and labelling with patient's name and hospital registration number, microcentrifuge tubes were stored in the freezer at − 80 °C until further ELISA investigation. ELISA was performed according to the manufacturer's instruction manual, and the colored product was read immediately at 450 nm wavelength. The excess sample was washed from the plate. Each sample was analyzed in duplicate for statistical analysis. MMP12 concentration in saliva was calculated with the help of standard curve using a known concentration of standard MMP12.

### Statistical analysis

Readings were obtained by using ELISA reader software. Data was analyzed using SPSS 23.0 version (SPSS, Inc., Chicago, IL). Pearson correlation test was applied to analyze the correlation between salivary MMP-12 level and age. Comparison of means among genders was tested by applying independent sample *t*-test. The statistical Analysis was run with a significance level of *p* < 0.05. One-way Analysis of variance (ANOVA) was run to determine if MMP-12 levels differ between healthy, OSF and OSCC groups, preceded by post hoc Tuckey test. The cut- off values of MMP-12 were determined by performing Receiver Operating Characteristic (ROC) curve analysis.

## Results

The socio-demographic data of all subjects included in the study is presented in Table 1. This data includes age, gender, marital status, employment status and oral habits of all participants.

**Table 1:** Sociodemographic data of patients

Category	Healthy n (%)	OSF n (%)	OSCC n (%)
Age			
(Mean S.D)	28.87 $\pm$ 6.81	33.27 $\pm$ 12.43	47.13 $\pm$ 13.38
Gender			
Male Female	5 (16.6%) 25 (83.3%)	25 (83.3%) 5 (16.6%)	20 (66.6%) 10 (33.3%)
Marital status			
Unmarried	8 (26.6%)	10 (33.3%)	1 (3.3%)
Employment Status			
Married	22 (73.3%)	20 (66.6%)	29 (96.6%)
Employed Unemployed	23 (76.6%) 7 (23.3%)	19 (63.3%) 11 (36.6%)	20 (66.6%) 10 (33.3%)
Oral habits			
Betel nut Tobacco smoking Smokeless tobacco None	7 (23.3%) 4 (13.3%) 0 (0%) 19 (63.3%)	17 (56.6%) 6 (20%) 7 (23.3%) 0 (0%)	10 (33.3%) 7 (23.3%) 4 (13.3%) 9 (30%)
Total	30	30	30

A statistical test was applied to evaluate the correlation between age and MMP-12 expression among healthy, OSF and OSCC group. A positively moderate correlation was observed between age and MMP-12 expression among the three groups (r = 0.35, *p* = 0.001). It demonstrates that as age increases, MMP-12 levels also increase.

A test was run to compare mean MMP-12 expression between gender. A highly statistically significant mean difference in MMP-12 expression was observed between gender (*p* < 0.001). Higher mean MMP-12 expression was found in Males (*M* = 12.5 ng/ml) compared to Females (*M* = 5.59 ng/ml). (Table 2).

**Table 2:** Mean comparison of Matrix metalloproteinases- 12 expressions with genders

Gender	N = 90	Mean (S.D)	M.D (*p* value ^ó^)
Male	50	12.5 (5.84)	6.91 (< 0.001)
Female	40	5.59 (6.72)	

^ó^- *p* value computed using Independent t- test

M.D- Mean difference, S.D- Standard Deviation

Oral habits of healthy, OSF and OSCC group were recorded. A statistical test was applied to compare the means of MMP-12 levels among participants with various oral habits. A statistically significant difference in means was observed (*p* < 0.05) among participants with distinct oral habits (Table 3).

**Table 3:** Descriptive ANOVA- MMP-12 expression & oral habits

Category	N = 90	Mean (SD)	MSE (*p* value ^ò^)
Betel nut	34	10.5^b^ (5.8)	44.46 (0.003^*^)
Tobacco smoking	17	10.5 (6.9)	
Smokeless tobacco	11	13.8^a^ (4.2)	
Non-significant oral habit	28	5.6^a,b^ (8.0)	

^Ò^—*p* value computed using One-way ANOVA

^*^- Significant at 0.05

MSE- Mean square error

^a^ on comparison p < 0.01

^b^ on comparison p < 0.05

The above table (Table 3) demonstrates a significant difference in mean between smokeless tobacco consumers and those with non-significant oral habits. MMP-12 levels among smokeless tobacco consumers appear higher as compared to participants with non-significant oral habits. A statistically significant difference in mean is also evident in betel nut consumers and participants with non-significant oral habits. Higher MMP-12 levels appear in betel nut consumers as compared to individuals with no significant oral habits.

The difference in mean MMP-12 level among healthy, OSF and OSCC group was analyzed. The result demonstrated a statistically significant difference in salivary MMP-12 means among the groups (*p* < 0.001) (Table 4).

**Table 4:** Descriptive ANOVA- MMP-12 expression (ng/ml)

Category	N = 90	Mean (SD)	MSE (*p* value ^ò^)
Healthy	30	0.82 (0.45)	12.48 (< 0.001)
OSF	30	12.53 (3.2)	
OSCC	30	14.92 (5.1)	

Comparison	Mean difference	*p* value ^ø^	
OSF vs Healthy	11.70	< 0.001	
OSCC vs Healthy	14.09	< 0.001	
OSCC vs OSF	2.39	0.028	

^**Ò**^- *p* value computed using One-way ANOVA

MSE- Mean square error, SD- Standard Deviation

^**ø**^- *p* value computed using Post hoc Tuckey test

Healthy participants demonstrated significantly different salivary MMP12 expression compared to patients belonging to Oral Submucous Fibrosis (*p* < 0.001) & Oral Squamous Cell Carcinoma (*p* < 0.001) group, respectively. Table 4 depicts the mean comparison of MMP-12 expression among the healthy, OSF, and OSCC groups at a statistically significant mean level (*p* = 0.028).

**Fig. 1:**
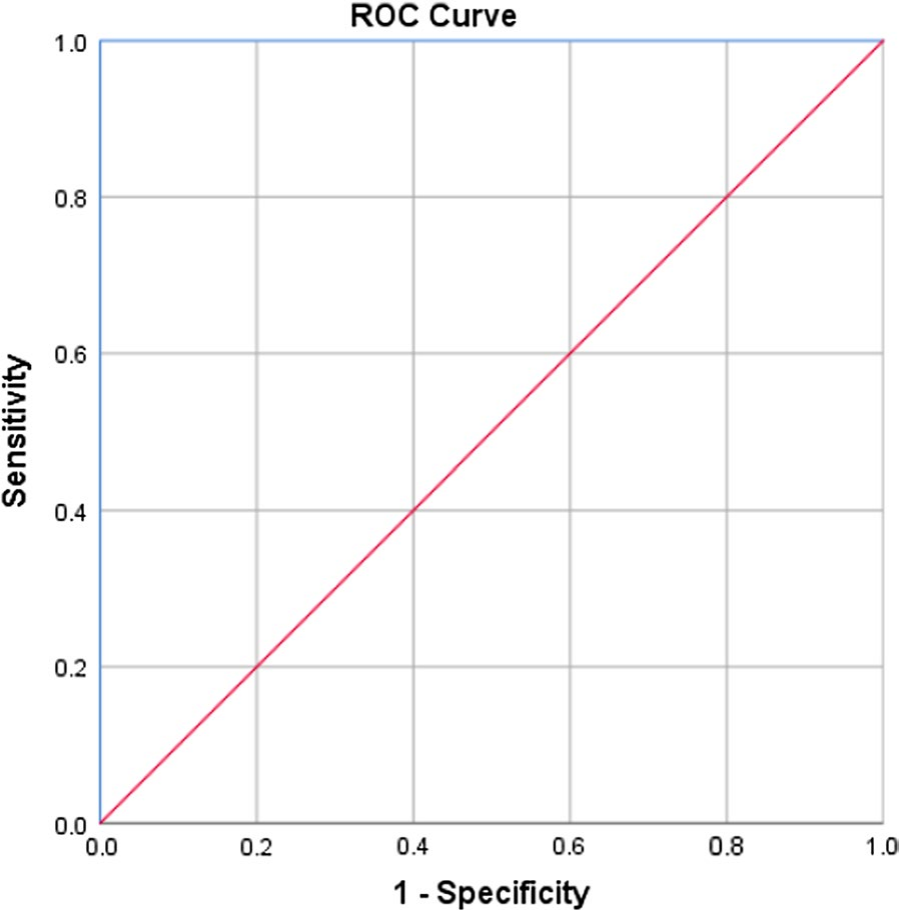
Receiver operating characteristics (ROC) curve analysis of the predictive value of MMP-12 for OSF

**Fig. 2:**
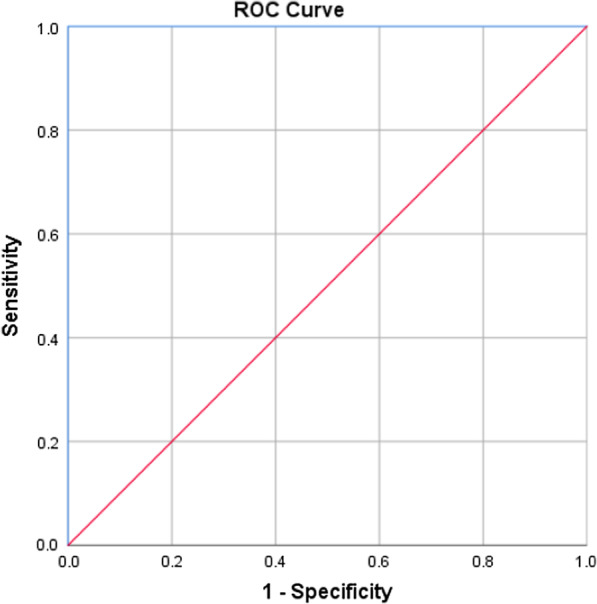
Receiver operating characteristics (ROC) curve analyses for predictive value of MMP-12 for OSCC

MMP-12 value of ≥ 4.06 ng/ml is predictive of Oral submucous fibrosis and MMP- 12 value of ≥ 4.21 ng/ml is predictive of Oral squamous cell carcinoma, with 100% sensitivity and 100% specificity (*p* < 0.001) (Table 5). The area under curve observed for OSF and OSCC is 100% (Figs. 1, 2).

**Table 5:** MMP12 Cut-off for OSF & OSCC using receiving operative curve (ROC)

Category	MMP12 Cut-Off	Sensitivity	Specificity	AUC (*p* value)
OSF	≥ 4.06	100%	100%	100% (< 0.001)
OSCC	≥ 4.21	100%	100%	100% (< 0.001)

AUC: Area Under the Curve

## Discussion

The level of MMP-12 appears to be highly expressed in a wide range of cancers, including colorectal, gastric, skin nasopharyngeal, and lung cancer [19–24]. A study was carried out to identify the expression of LIFR, PIK3R1, and MMP- 12 in gall bladder carcinoma. This study validated MMP-12 as a significant prognostic biomarker in this rare and aggressive tumor [25]. Another study was conducted to analyze the expression of MMP-12 level in patients presenting with laryngeal squamous cell carcinoma. There was a poorer degree of tumor differentiation as the expression of MMP-12 went higher [26]. Elevated levels of MMP-12 were also correlated with pathological stage and metastasis of lung adenocarcinoma. Hence, targeting MMP-12 for the treatment of lung adenocarcinoma seemed promising. In addition to this, high expression of MMP-12 was observed in esophageal squamous cell carcinoma compared to normal epithelial cells [27]. Due to its functional properties and its role in tissue destructive disease, MMP 12 can be used as a biomarker for various oral diseases [28]. It plays a significant part in tumorigenesis and progression. This includes tumor growth, migration, invasion and tumor metastasis [15, 29, 30]. MMP-12 is recommended as a diagnostic biomarker for OSCC due to its significant sensitivity and specificity [15].

The current study consisted of participants with a minimum age of 18 years and maximum age of 78 years with a mean age of 36.42 $\pm$ 13.60 years. Statistical Analysis was applied to observe the relationship between salivary MMP-12 expression and participants' age. The results demonstrated a statistically significant direct relationship between the two variables, which explains that MMP-12 expression in saliva increases as age increases. In contrast to our findings, lower salivary MMP-12 levels were reported in a study in individuals aged 40–60 years compared to individuals under 40 years [28].

A higher proportion (56%) of research participants were males, whereas 44% were females. A statistically significant difference was observed in salivary MMP-12 expression among genders, implying a higher mean expression of salivary MMP-12 in males than females. However, no difference in salivary MMP-12 was observed between males and females in a study focusing on MMP-12 levels in patients with periodontal inflammation [28].

The study also illustrates the oral habits of participants. Majority of the participants were betel nut consumers, followed by patients reporting tobacco smoking as their oral habit. A comparison was made between distinct oral habits and salivary MMP-12 expression. A significant difference was observed between MMP-12 expression of individuals with distinct oral habits. Smokeless tobacco consumers demonstrated higher MMP-12 expression compared to other oral habits. However, individuals who reported tobacco and betel nut as their oral habit demonstrated lower MMP-12 expression comparatively. In contrast to the results mentioned, another study evidences a non-significant difference in MMP-12 expression among patients with different oral habits, including smoking, alcohol, and betel nut chewing [31].

The current study results demonstrated a statistically significant difference in salivary MMP-12 expression in OSF and OSCC group compared to healthy participants. OSF and OSCC group showed higher salivary MMP-12 expression, and lower expression was observed in healthy group. The results coincide with a previous study that indicated a high association of salivary protease spectrum with oral health status [17]. It demonstrated increased protease levels in OSCC patients as compared to patients presenting with other oral diseases. MMP- 12 was detected only in the saliva of patients with OSCC along with other MMPs, such as MMP-1, MMP-2, MMP-3, MMP-10, and MMP-13. In addition to this, MMP-1, MMP-2, MMP-10, and MMP-12 were also observed to be significantly increased in patients with OSCC compared to healthy patients and patients with benign oral masses (OBM) and mild chronic periodontitis (CPD). The concentration of salivary MMP-12 in OSCC patients demonstrated in this study is around 1300 pg ml^−1^, which is comparatively more than healthy (700 pg ml^−1^), benign oral mass (900 pg ml^−1^), and chronic periodontal disease patients (900 pg ml^−1^) [17].

A cohort study carried out in Sweden on 436 participants aimed to investigate salivary MMP-12 levels to various aspects of oral health. The influence of non-disease covariates on MMP-12 levels was also assessed. The results revealed an association between MMP-12 levels and the percentage of gingival pockets $\ge$ 4 mm. The study concluded that MMP-12 reflects various aspect of periodontal disease and the levels are contrarily affected by the presence of tumor [28]. The results corroborate the results of our study in which MMP-12 levels are affected in the presence of tumor.

A study was conducted to compare the MMP12 level in patients presenting with OSCC and verrucous carcinoma (VC) in tissue samples. The study results showed that VCs were devoid of epithelial MMP-12 expressions compared to SCC [32]. Another study was undertaken to estimate serum MMPs levels in OSCC patients compared to healthy participants. Serum level of MMP-12 was notably arisen in OSCC patients as compared to healthy participants [15].

MMP-12 expression found elevated in patients with chronic periodontitis with identification of CD68+ CD14+ CD64+ cells [28]. Also, the expression of MMP 12 in tissue samples appear high in patients with extracapsular spread compared to those without extracapsular spread [24]. Hence, it may be a useful predictive marker for extracapsular spread (ECS) in head and neck tumors [24].

In recent years, the prevalence of OSF has increased from 8.3/10^5^ to 16.2/10^5^ [33, 34]. The rate of malignant transformation to oral cancer is 9.13%, and there is a 29.26 times higher risk in OSF patients than non-OSF patients [35, 36]. In Pakistan, OSF is contemplated as a public health concern as oral malignancies are one of the most common malignancies reported [37]. Also, oral habits like tobacco smoking and consumption of betel nut and smokeless tobacco are major risk factors of OSF and are common in Pakistan. Studying the role of several markers present in saliva will help in devising a non-invasive investigation for OSF diagnosis, which will ultimately result in early diagnosis of OSF and prevent it from advancing to OSCC, if treated promptly. Since OSF is an oral potentially malignant disorder and is common in our part of the world, we have studied the expression of MMP-12 in OSF patients along with OSCC patients.

Statistically, a significant difference in salivary MMP-12 level was observed in OSF patients in comparison with healthy participants. OSF patients demonstrated higher MMP-12 levels. However, in comparison with salivary MMP-12 in OSCC, salivary MMP-12 in OSF demonstrated lower expression explaining the increase in MMP-12 expression as an oral potentially malignant disorder (OSF) drifts towards malignancy (OSCC). Since the significant difference in salivary MMP-12 levels is observed between OSF and OSCC, this investigation may serve as a beneficial non-invasive tool for differentiating OSCC from specifically last stage OSF in which patients present with nil mouth opening and the surgeon suspects a lesion intraorally.

## Conclusion

The current study indicates salivary MMP-12 expression in patients presenting with oral submucous fibrosis and biopsy-proven oral squamous cell carcinoma. We observed a statistically significant difference in salivary MMP-12 expression in OSF and OSCC group as compared to healthy patients, with higher expression in OSF and OSCC patients. The study results also demonstrate higher expression of salivary MMP-12 in OSCC as compared to OSF. Therefore, the estimation of salivary MMP-12 may serve as a useful non-invasive early diagnostic tool in diagnosing oral submucous fibrosis and oral squamous cell carcinoma.

## Data Availability

The datasets used and analyzed during the current study are available from principal investigator (corresponding author) on reasonable request.

## Abbreviations

OSCC: Oral squamous cell carcinoma
OSF: Oral submucous fibrosis
MMP: Matrix metalloproteinases
ELISA: Enzyme-linked immunosorbent assay
ANOVA: One-way analysis of variance
OBM: Oral benign mass
CPD: Mild chronic periodontitis
VC: Verrucous carcinoma
ROC: Receiver operating characteristics
SD: Standard deviation
MD: Mean difference
MSE: Mean square error
